# Exercise Modulates Oxidative Stress and Inflammation in Aging and Cardiovascular Diseases

**DOI:** 10.1155/2016/7239639

**Published:** 2015-12-28

**Authors:** Nada Sallam, Ismail Laher

**Affiliations:** ^1^Department of Pharmacology and Toxicology, Faculty of Pharmacy, Cairo University, Cairo 11562, Egypt; ^2^Department of Pharmacology and Therapeutics, Faculty of Medicine, The University of British Columbia, Vancouver, BC, Canada V6T 1Z3

## Abstract

Despite the wealth of epidemiological and experimental studies indicating the protective role of regular physical activity/exercise training against the sequels of aging and cardiovascular diseases, the molecular transducers of exercise/physical activity benefits are not fully identified but should be further investigated in more integrative and innovative approaches, as they bear the potential for transformative discoveries of novel therapeutic targets. As aging and cardiovascular diseases are associated with a chronic state of oxidative stress and inflammation mediated via complex and interconnected pathways, we will focus in this review on the antioxidant and anti-inflammatory actions of exercise, mainly exerted on adipose tissue, skeletal muscles, immune system, and cardiovascular system by modulating anti-inflammatory/proinflammatory cytokines profile, redox-sensitive transcription factors such as nuclear factor kappa B, activator protein-1, and peroxisome proliferator-activated receptor gamma coactivator 1-alpha, antioxidant and prooxidant enzymes, and repair proteins such as heat shock proteins, proteasome complex, oxoguanine DNA glycosylase, uracil DNA glycosylase, and telomerase. It is important to note that the effects of exercise vary depending on the type, intensity, frequency, and duration of exercise as well as on the individual's characteristics; therefore, the development of personalized exercise programs is essential.

## 1. Exercise Training and Aging

There is mounting evidence based on epidemiologic and experimental studies that physical activity and exercise training combat the sequels of aging. Physical activity is defined as any bodily movement coordinated by skeletal muscles, which increases energy expenditure over resting condition [1], whereas exercise training is a more regular and structured form of physical activity. Higher levels of physical activity and regular exercise are associated with reduced risks of all-cause mortality [2–20] and also with increased longevity [18, 21–23]. In fact, the World Health Organization has identified physical inactivity as the fourth leading risk factor for global mortality [24]. Furthermore, physical activity and exercise training reduce the risk of age-associated diseases, namely, cardiovascular diseases [4, 10, 25–31], type 2 diabetes [32], metabolic syndrome [33], colon cancer [34], obesity [35], osteoporosis [36], sarcopenia [37], anxiety [38], and cognitive impairment [39–41]. Most importantly, exercise improves the quality of life of elderly people [42, 43].

## 2. Exercise Training and Cardiovascular Diseases

Age is a major risk factor for cardiovascular diseases (CVDs) [44, 45]. Numerous studies, confirmed by meta-analyses, indicate that exercise training reduces cardiovascular mortality [7, 20, 26, 46–54] and cardiovascular events [4, 10, 25–28, 30, 31], particularly stroke [55–58], coronary heart disease [25, 59–61], heart failure [60, 62, 63], atherosclerosis [64–66], and preeclampsia [67–69]. Accordingly, physical inactivity is now regarded as one of the most prevalent cardiovascular risk factors [70, 71]. Moreover, exercise training is an effective therapeutic strategy for patients with peripheral arterial diseases [72–74], coronary heart disease [75–81], heart failure [82–84], atherosclerosis [64], and hypertension [85–88].

The cardiovascular benefits of exercise have been frequently attributed to the reduction of many classical cardiovascular risk factors including blood lipids [20, 28, 50, 89–95], high blood pressure [20, 28, 50, 95], obesity [50, 95–97], glucose, and type 2 diabetes [98, 99] as well as novel risk factors such as inflammation [28, 100–103] and oxidative stress [95]. However, the mechanisms underlying the protective and therapeutic effects of exercise go beyond reducing cardiovascular risk factors [104] to modulating angiogenesis [105], endothelial progenitor cells [106–109], basal heart rate [110], endothelial function [111–115], autonomic control [116], arterial stiffness [41, 117–120], and arterial remodeling [121]. In this review we will focus on the molecular transducer of the antioxidant and anti-inflammatory effects of exercise.

## 3. Oxidative Stress and Inflammation in Aging and Cardiovascular Diseases

Aging is associated with oxidative stress that is mainly attributed to defective mitochondria, resulting from reduction in cytochrome C oxidase (complex IV) activity [23, 122, 123] and peroxidative damage of mitochondrial membrane [124]. Hence, a greater number of electrons are generated that can escape from the mitochondria to create a long trail of reactive oxygen species (ROS) [125, 126], leading to further mitochondrial dysfunction and ROS generation and creating a vicious cycle of oxidative damage [127]. Age-associated increases in ROS production occur in skeletal muscles [128] and other organs such as the heart, liver, brain, and kidney [23, 126, 129, 130].

Reduced protein synthesis limits antioxidant defense mechanisms and repair capacity in aged individuals, which further contributes to the state of oxidative stress. The free radical theory of aging hypothesizes that oxidative stress damages macromolecules, including lipids, proteins, and nucleic acids, overwhelming cellular antioxidant defense and repair mechanisms, leading to progressive deleterious changes over time [131, 132]. Indeed, oxidatively damaged proteins [133], nucleic acids [134, 135], and lipids [113, 136–138] are abundant in various organs and tissues such as kidney, liver, heart, arteries, skeletal muscles, and plasma in aged subjects.

Aging is also accompanied with a state of chronic inflammation that is mainly attributed to sarcopenia and adiposity. Sarcopenia, defined as age-associated progressive loss of muscle mass and strength [139, 140], increases the incidence of muscle injury [18], which increases the infiltration of immune cells into injured muscles. Activated immune cells and injured muscles release proinflammatory mediators and reactive oxygen and nitrogen species (RONS) via lipoxygenase, NADPH oxidase, xanthine oxidase, and inducible nitric oxide synthase (iNOS) [141–147] leading to oxidative stress.

Sarcopenia can also lead to reduced physical activity and increased adiposity. Adiposity induces a state of low-grade but chronic inflammation through the release of a multitude of proinflammatory cytokines including tumor necrosis factor-alpha (TNF-*α*), interleukin-6 (IL-6), and interleukin-1 beta (IL-1*β*) [148–150]. Indeed, aging is associated with increased levels of TNF-*α*, IL-6, and interleukin-1 receptor agonist (IL-1ra) and systemic inflammatory biomarkers such as C-reactive protein (CRP) as well as higher count of inflammatory cells such as neutrophil and monocytes [151–153]. Hence, aging is associated with oxidative stress and inflammation.

Cardiovascular diseases are also associated with high level of inflammation and oxidative stress [154–157].

## 4. Oxidative Stress and Inflammation Overlapping Signaling Pathways

Oxidative stress and inflammation share common and overlapping signaling pathways. By damaging macromolecules, ROS can initiate inflammation [158]; ROS are also products of the inflammatory process. During the respiratory burst, immune cells generate RONS via NADPH oxidase and iNOS and release proinflammatory cytokines such as TNF-*α,* IL-1*β*, and IL-6 [141, 159, 160]. Similarly, injured tissues can also release proinflammatory cytokines that activate specific ROS-generating enzymes such as lipoxygenase, NADPH oxidase, myeloperoxidase, and xanthine oxidase [142–147] and specific reactive nitrogen species generating pathways such as NOS, protein kinase B (Akt), and Sph1P (sphingosine-1-phosphate) [161–163].

ROS overproduction activates redox-sensitive transcription factors including nuclear factor kappa B (NF-*κ*B) and activator protein-1 (AP-1) via stress kinases such as extracellular signal regulated kinases (ERKs), c-jun N-terminal kinases (JNKs), mitogen activated protein kinase p38 (MAPK p38), protein kinase C (PKC), phosphatidylinositol-4,5-bisphosphate 3-kinase (PI3K)/Akt, and Src family kinases (SFKs). This leads to increased expression of inflammatory target proteins such as matrix metalloproteinase-9 (MMP-9), intercellular adhesion molecule-1 (ICAM-1), vascular cell adhesion molecule-1 (VCAM-1), iNOS, cyclooxygenase-2 (COX-2), and cytosolic phospholipase A2 (cPLA2) (Lee and Yang) [164–171] and proinflammatory mediators such as TNF-*α* gene [172], IL-1, and IL-8 [169]. Many of these inflammatory proteins or their products such as NOS, COX, and PGE_2_ are prominent sources of RONS [173]; this creates an autoactivating loop which feeds the vicious cycle of inflammation and oxidative stress.

There are also other proteins such as thioredoxin-interacting protein (TXNIP) linking oxidative stress and inflammation. Under resting conditions, TXNIP is bound to thioredoxin (TRX) via a disulphide bound, keeping it in an inactive form. Increased levels of ROS generation cause the dissociation of TXNIP from TRX, leaving it free to scavenge ROS and allowing TXNIP to stimulate the inflammatory cytokine IL-1*β* [174, 175]. In agreement with this is the observation that antioxidant supplementation blocked the anti-inflammatory effect of exercise by reducing IL-6 production [176, 177].

**Figure 1:**
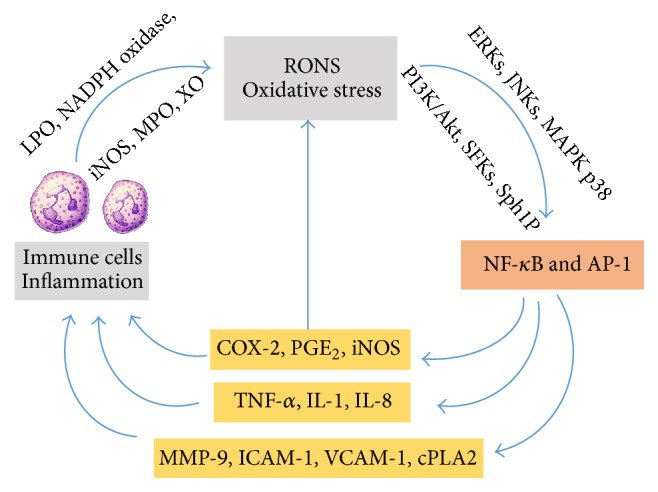
Oxidative stress and inflammation overlapping signaling pathways in aging. AP-1 = activator protein-1, COX-2 = cyclooxygenase-2, cPLA2 = cytosolic phospholipase A2, ERKs = extracellular signal regulated kinases, ICAM-1 = intercellular adhesion molecule-1, IL-1 = interleukin-1, IL-8 = interleukin-8, iNOS = inducible nitric oxide synthase, JNKs = c-jun N-terminal kinases, LPO = lipoxygenase, MAPK p38 = mitogen activated protein kinase p38, PI3K = phosphatidylinositol-4,5-bisphosphate 3-kinase, MMP-9 = matrix metalloproteinase-9, MPO = myeloperoxidase, NF-*κ*B = nuclear factor kappa B, PGE_2_ = prostaglandin E_2_, PKC = protein kinase C, RONS = reactive oxygen nitrogen species, Sph1P = sphingosine-1-phosphate, TNF-*α* = tumor necrosis factor-alpha, VCAM-1 = vascular cell adhesion molecule-1, and XO = xanthine oxidase.

In short, proinflammatory mediators such as TNF-*α*, IL-1, and IL-6 generate RONS which activate redox-sensitive transcription factors such as NF-*κ*B and AP-1 resulting in the generation of large quantities of these proinflammatory mediators and ROS (Figure 1). Indeed, aging is associated with adverse health conditions such as atherosclerosis, metabolic syndrome, sarcopenia, arthritis, and chronic obstructive pulmonary disease that are characterized by elevated levels of both oxidative stress and inflammatory markers [178].

Not surprisingly, ROS can also induce proteins such as heat shock proteins (HSPs), HSP70 in particular [179], and heme oxygenase 1 (HO-1) [180] that can protect cells and tissues from the deleterious effects of inflammation. However, the balance of antioxidant/anti-inflammatory to oxidant/inflammatory proteins is tilted towards the latter during the aging process.

## 5. Exercise Training: Modulation of Oxidative Stress and Inflammation

**Figure 2:**
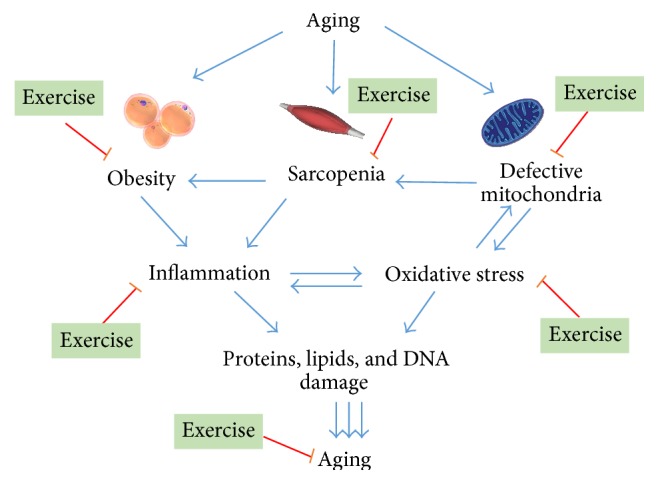
Modulation of oxidative stress and inflammation in aging by exercise.

Exercise and regular physical activity counteract the deleterious effects of aging, not only by combating sarcopenia, obesity, and mitochondrial dysfunction, the major triggers of oxidative stress and inflammation in aging, but also by exerting additional antioxidant and anti-inflammatory actions as illustrated in Figure 2.

### 5.1. Effect of Exercise Training on Adiposity

Adipose tissue, particularly visceral fat depots, and the macrophages trapped within fat depots are able to release proinflammatory cytokines such as IL-6 and TNF-*α* [148–150, 181, 182]. Physical activity and exercise training increase energy expenditure and reduce body fat, particularly visceral fat, with/without weight loss [35, 183, 184], and therefore reduced production and release of IL-6 and TNF-*α* [185–189]. Exercise training increased gene expression of PGC-1 alpha, a master regulator of mitochondrial biogenesis, in rat adipose tissue [190], leading to increased energy expenditure particularly in the visceral area. Exercise training inhibited the infiltration of the inflammatory phenotype M1 macrophages into adipose tissue, while also favoring the switch of macrophages to the less inflammatory phenotype M2 in obese mice [191]. Exercise training/physical activity also induces the release of adiponectin from adipose tissues [192–197]; adiponectin exerts antiapoptotic, anti-inflammatory, and antioxidative activities [198, 199].

### 5.2. Effect of Exercise Training on Skeletal Muscles

Physical activity/exercise increases nutritive blood supply to and removes waste from skeletal muscles, while also upregulating the expression of the anabolic myokine IL-15 [195–197, 200, 201]. Most importantly, physical activity/exercise stimulates mitochondrial biogenesis [202] and oxidative capacity [203] that provide energy for the synthesis of new proteins. Thus, physical activity/exercise improves muscle mass and strength and renders them less vulnerable to acute injury [204], therefore suppressing triggers of inflammation and oxidative damage [205–209].

Physical activity/exercise also induces the release of several myokines from skeletal muscle such as IL-6 [210–212], which suppresses IL-1 and TNF-*α* [213] and triggers the release of many anti-inflammatory cytokines such as IL-1 receptor antagonist (IL-1ra) and IL-10, in addition to cortisol [214, 215]. In turn, IL-10 inhibits the synthesis of some proinflammatory cytokines such as TNF-*α* and IL-1*β* [200]. Exercise also reduces TNF-*α* and IL-1*β* production in skeletal muscles [212, 216, 217].

Heat shock proteins (HSPs) are also generated in skeletal muscles in response to physical activity/exercise; they exert vital anti-inflammatory action as will be explained later [218–222].

### 5.3. Effect of Exercise Training on Mitochondrial Aging

Exercise mitigates mitochondrial aging and interrupts the vicious cycle of oxidative damage by stimulating mitochondrial biogenesis [202] and enhancing mitochondrial oxidative capacity [203, 223, 224]. Excellent reviews on this topic are available [225, 226].

### 5.4. Anti-Inflammatory Effects of Exercise Training

Acute bouts of exercise cause transient damage to contracting skeletal muscles, triggering an inflammatory response that increases the levels of proinflammatory cytokines and acute-phase reactants in the blood [227–230]. However, regular exercise reduces levels of systemic inflammatory markers such as CRP, IL-6 TNF-*α*, soluble TNF-*α* receptor 1 (sTNF-R1), and soluble TNF-*α* receptor 2 (sTNF-R2) in young and middle aged adults [231–243] and also more importantly in the elderly [195, 244–250]. Additionally, higher levels of the anti-inflammatory cytokines interleukin-10 (IL-10) [246] and adiponectin [195] are associated with increased physical activity in the elderly. Several interventional studies report that exercise reduces inflammatory markers, particularly CRP, TNF-*α*, interferon-gamma (INF-*γ*), monocyte chemoattractant protein-1 (MCP-1) and interleukin-8 (IL-8), interleukin-18 (IL-18), sTNFR2, sTNF-R1, and soluble IL-6 receptor (sIL-6R), and increases levels of anti-inflammatory factors such as IL-10, interleukin-12 (IL-12), interleukin-4 (IL-4), and transforming growth factor beta 1 (TGF*β*1) [74, 188, 197, 217, 251–265]. These benefits of exercise also occur in the elderly [100, 196, 250, 263, 266–271] as summarized in Table 1. However, only a few randomized controlled trials confirm the anti-inflammatory effect of exercise [74, 261, 262, 269]. Exercise training can exert anti-inflammatory effects with/without accompanied weight loss; however, the most substantial anti-inflammatory effects occur in patients with high baseline inflammatory biomarkers, particularly when associated with weight loss [260].

It is worth noting that some interventional and randomized controlled trials studies did not detect a significant effect of regular exercise on systemic inflammatory biomarkers in adults [243, 272–274] or in aged adults [275–278] as shown in Table 1. A meta-analysis found only five randomized controlled trials that examined the effects of regular aerobic exercise (of at least 4-week duration) in adults and concluded that aerobic exercise did not reduce CRP levels [279]. It is likely that these discrepancies may be attributed to the smaller sample size used in the clinical trials examined.

On the other hand, the effects of resistance exercise on inflammatory mediators are mostly negative [280–282], although Brooks et al. [196] reported that 16 weeks of resistance training reduced CRP and increased adiponectin levels in older diabetic patients. The effects of physical activity and different exercise programs on inflammatory mediators in the elderly are detailed in Table 1. Clearly, the effects of exercise depend on the type (aerobic/resistance), intensity (mild/moderate/intense/exhaustive), and frequency (sessions per day/week/month) of exercise and also on the subject's characteristic (age, sex, endurance capacity, and health condition).

#### 5.4.1. Molecular Transducer of the Anti-Inflammatory Effects of Exercise Training

The signaling pathways underlying the anti-inflammatory effects of exercise are complex and not completely understood. In addition to the effects of exercise on adipose tissue, skeletal muscles, and mitochondrial biogenesis mentioned above, exercise exerts additional anti-inflammatory actions on the immune system, repair mechanisms, and vasculature.

*Effects of Exercise on the Immune System.* Regular exercise downregulates the innate immune response and activates the adaptive immune system with consequent suppression of inflammation. Exercise modulates the immune system by reducing the number of inflammatory CD14+CD16+ monocytes [250], increasing the number of CD4CD25 regulatory T cells [264, 283], shifting blood macrophages towards the less inflammatory phenotype M2 [284], increasing the dominance of the anti-inflammatory Type 2 helper T cell over proinflammatory Type 1 helper T cell [265, 284–286], and reducing monocyte chemoattractant protein-1 (MCP-1) [188] and toll-like receptor-4 (TLR4) expression on monocyte surfaces [239, 263, 275]. On the other hand, ET increases the production of transforming growth factor beta (TGF*β*1) [254, 264] from regulatory T cells.

Exercise also stimulates the sympathetic nervous system and the hypothalamic-pituitary-adrenal axis to increase serum glucocorticoid levels [287] with subsequent inhibition of the immune system [288].

*Effects of Exercise on Repair Mechanisms.* Heat shock proteins are highly conserved chaperone proteins that regulate the folding and processing of damaged proteins and therefore exert significant anti-inflammatory action. Numerous studies have shown that exercise is capable of upregulating the expression of HSP72 [219, 222, 289–293], HSP70 [137, 218, 220, 221, 294–297], HSP60 [294, 298], HSP27 [294, 295], and HSP25 [222] in skeletal muscles, blood cells, hearts, and arteries of humans as well as young and aged experimental animals (see Table 2). However, Hägg et al. [299] reported that voluntary wheel running of spontaneously hypertensive rats for 5 weeks reduced aortic gene expression of HSP70 and HSP60. The effect of exercise on HSPs depends on age [296], sex [220], time course [221, 291], and HSP subtype [222, 293, 294, 298].

**Figure 3:**
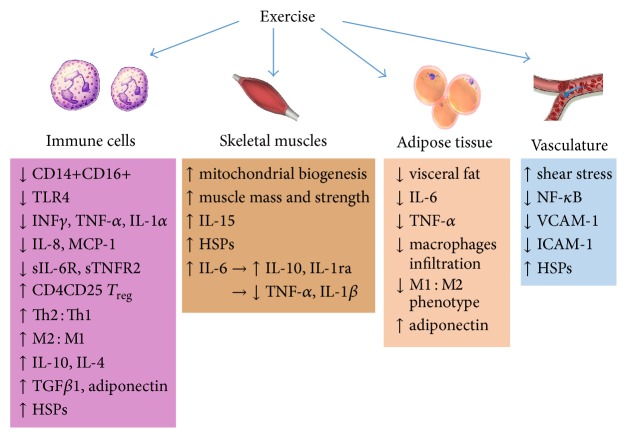
Signaling pathways underlying the anti-inflammatory actions of exercise. HSPs = heat shock proteins, IL-1*α* = interleukin-1-alpha, IL-1ra = interleukin-1 receptor antagonist, IL-1*β* = interleukin-1 beta, IL-6 = interleukin-6, IL-8 = interleukin-8, IL-10 = interleukin-10, IL-15 = interlukin-15, INF*γ* = interferon gamma, M1 = macrophage phenotype 1, M2 = macrophage phenotype 2, ROS = reactive oxygen species, sTNFR2 = soluble TNF-*α* receptor 2, sIL-6R = soluble IL-6 receptor, TLR4 = toll-like receptor-4, TGF*β*1 = transforming growth factor beta 1, TNF-*α* = tumor necrosis factor-alpha, Th1 = Type 1 helper T cell, and Th2 = Type 2 helper T cell.

*Effects of Exercise on Vascular Endothelial Cells.* By increasing shear stress on vascular endothelial cells, exercise modulates key players in the inflammatory process such as ICAM-1, NF-*κ*B, MAPK, and COX-2 [300, 301]. Voluntary wheel running of aged mice for 10–14 weeks reduced the activation of NF-*κ*B in the aorta [302]. Subjecting aortic endothelial cells to* in vitro* shear stress for 4 h reduced the expression of vascular cell adhesion molecule-1 (VCAM-1) [303]. Treadmill training for 1–3 weeks reduced the expression of intercellular cell adhesion molecule-1 (ICAM-1) in response to cerebral ischemia in rats [304]. The mechanisms underlying the anti-inflammatory actions of exercise are summarized in Figure 3.

### 5.5. Antioxidant Effects of Exercise Training

Generation of ROS is transiently increased during exercise; however, the incidence of diseases associated with oxidative stress is reduced by regular exercise. Regular exercise attenuates oxidative damage in the brain [23, 305–307], liver [23, 130, 308–310], kidney [23, 136], skeletal muscle [311], blood [113, 136], and heart [23, 297]. However, Goto et al. [135] found that high-intensity exercise for 12 weeks increased the indices of oxidative stress in young men.

Importantly, regular exercise ameliorates age-associated oxidative stress in the heart [297, 312], liver [130], plasma [113], arteries [138], and skeletal muscles [313, 314]. In the study of Navarro et al. [23], exercise reduced age-associated mitochondrial oxidative damage and upregulated mitochondrial NADH-cytochrome C reductase and cytochrome oxidase activities in brain, heart, liver, and kidney of 52-week-old but not older rats. However, exercise caused an increase in oxidative damage in skeletal muscles [315] and hearts of aged rats [316].

In elderly people, regular exercise reduced serum/plasma levels of myeloperoxidase, a marker of inflammation and oxidative stress [205], and thiobarbituric-reactive acid substances, a marker of lipid peroxidation [317]. Lower levels of nitrotyrosine [133] and thiobarbituric-reactive acid substances [318] were found in the more physically active elderly people. However, de Gonzalo-Calvo et al. [319] reported that although regular exercise increased protein carbonyl content and lipid peroxidation levels in the plasma and erythrocytes of long term trained elderly men, their overall health condition was markedly improved. Another clinical study showed that 8 weeks of walking exercise did not significantly change low density lipoprotein (LDL) oxidation or nitration in the elderly [320].

#### 5.5.1. Molecular Transducer for the Antioxidant Effects of Exercise Training

As discussed above, exercise exerts prominent anti-inflammatory actions, thus suppresses major sources of ROS and RNS generation, and produces indirect antioxidant effects. Exercise also upregulates the antioxidant defense mechanisms and repair proteins in the body via redox-sensitive transcription factors, mainly NF-*κ*B, activator protein-1 (AP-1) and peroxisome proliferator-activated receptor gamma coactivator 1-alpha (PGC-1*α*), and by increasing laminar shear stress on vascular endothelial cells.

The metabolic demands of skeletal muscles increase during exercise; the body responds by increasing oxygen uptake and blood flow to the muscles and other body organs. The increased metabolic rate results in greater ROS production in skeletal muscles [128, 321] and in other organs as well [129, 322]. Sources other than the electron transport chain enzymes in the mitochondria, such as xanthine oxidase [323–325] and NADPH oxidase [128, 326], contribute to ROS generation during exercise. This transient increase in ROS levels activates NF-*κ*B, AP-1, and PGC-1*α* signaling.

*Effects of Exercise on NF-κB and AP-1 Signaling.* Exercise-induced increase in ROS levels triggers an adaptive antioxidant response that is mediated via mitogen activated protein kinases (MAPK p38, ERK 1, and ERK 2) [323, 327–329], cAMP-response-element binding (CREB) [330, 331], and synapsin [330, 331], to activate redox-sensitive transcription factors such as NF-*κ*B [323, 332, 333] and AP-1 [327, 333], resulting in increased expression of antioxidant enzymes [334] such as superoxide dismutase (SOD) [323, 333] and catalase [333], repair proteins such as hear shock proteins HSP25, HSP60, HSP72, HSP70, and heat shock cognate 70 HSC70 [315, 333–336], proteasomes complex, and nitric oxide synthase (NOS) [308, 323]. These signaling cascades were demonstrated in skeletal muscles [323, 332], brain [330, 331], leukocytes [337], and hearts [327] of experimental animals as well as in humans [328, 337] and in aged animals [333, 335, 338] and elderly people [337]. However, other studies report that exercise-induced activation of NF-*κ*B and AP-1 [333] and upregulation of HSP70 were attenuated in fast skeletal muscles of old rats [339]. Interestingly, aging also increased ROS production and NF-*κ*B activity in the livers of aged rats; these effects were attenuated by exercise [130, 308].

*Effects of Exercise on PGC-1α Signaling.* Exercise stimulates mitochondrial biogenesis [202] and ameliorates the age-associated decline in mitochondrial oxidative capacity in skeletal muscles [223] and other organs [23, 190, 340] via PGC-1*α* signaling [190, 341, 342]. PGC-1*α* is a redox-sensitive transcription factor that is activated by 5′-AMP-activated protein kinase (AMPK) [329, 343–345] to trigger the transcription of nuclear respiratory factor 1 (NRF-1) and expression of mitochondrial transcription factor A (mtTFA), a key regulator of mitochondrial DNA replication [346]. PGC-1*α* also increases the expression of antioxidant proteins such as glutathione peroxidase (GPX) and SOD-2 [347]. Safdar et al. [348] report that exercise reversed most of the multisystem pathology and premature mortality in mice which were genetically modified to accumulate mitochondrial mutations. The effects of exercise on AMPK and PGC-1*α* were preserved in the hippocampus of aging rats. However, results from Derbré et al. [341] suggest a blunted effect of exercise response in PGC-1*α* and NRF-1 in skeletal muscles of aged rats.

*Effects of Exercise on Vascular Endothelial Cells.* To meet the increasing metabolic demands of the body during exercise, perfusion of skeletal muscles and other tissues increases, subjecting vascular endothelial cells to higher levels of laminar shear stress. Increased laminar shear stress modulates gene expression and activity of SOD [349–351] possibly via NF-*κ*B and MAPK signaling [300, 301].

*Effects of Exercise on Antioxidant and Prooxidant Enzymes Expression and Activity.* The NF-*κ*B, AP-1, PGC-1*α*, and shear stress signaling cascades converge to upregulate antioxidant defense mechanisms to counteract and interrupt the vicious cycle of inflammation and oxidative stress associated with aging and cardiovascular diseases. The most intensely studied antioxidant enzymes in laboratory animals and in humans are SOD, catalase, GPx, glutathione transferase (GST), and glutathione reductase (GSR). The effects of exercise on antioxidant enzymes are summarized in Table 3.

Athletes' erythrocytes had higher SOD activity compared with untrained individuals [352, 353]. Regular and acute exercise increased SOD activity in erythrocytes of men and women [354, 355] and heart [23, 136, 292, 293, 316, 356–358], lung [23, 356], kidney [23, 136], brain [23], skeletal muscles [136, 311, 358], and arteries [359, 360] of experimental animals, particularly of aged animals [311, 316, 356, 358, 360].

*(1) SOD-1.* Just increasing* in vitro* shear stress is sufficient to upregulate the gene and protein expression of SOD-1 in human endothelial cells [349–351]. A study by Ennezat et al. [361] showed that regular exercise for 12 weeks increased gene expression of SOD-1 in skeletal muscles of congestive heart failure patients. Increases in protein expression of SOD-1 were observed after exercise in skeletal muscles [222, 362], heart [295, 363], brain [307], and arteries [194, 364, 365] of several experimental animals, importantly of aged animals [295]. However, other studies reported decreased or no change in expression of SOD-1 following long term exercise in aged animals [222, 366] and adult animals [367].

*(2) SOD-2.* Increases in activity have been observed in heart [230, 292, 296, 297, 368–370], skeletal muscles [371], plasma [137], and liver [372] of experimental animals following exercise. Increased protein expression has been reported in plasma and vascular endothelial cells of humans [133, 373] and in heart [230, 295, 296, 363, 374], skeletal muscles [362, 371, 375], liver [372], and arteries [138, 376] of experimental animals, notably in aged animals [138, 295, 296, 375].

*(3) SOD-3.* Higher activity was found in more physically active older men [133] and increased protein expression was observed in men after a bout of acute exercise but not endurance training [373]. However, increased protein levels were detected in experimental animals after endurance exercise [366, 367].

*(4) Catalase.* Winter swimmers had higher catalase activity in their erythrocytes than untrained subjects [353], while sprint-trained athletes exhibited lower activity than controls [352]. Exercise increased catalase activity in brain [23, 377, 378], heart [23, 136, 297, 316, 370], lung [377], skeletal muscles [136, 371, 377], liver [23, 126, 136, 356, 377], kidney [23], and cardiac mitochondria [379] of experimental animals and importantly in aged animals [126, 297, 316, 356, 377, 378]. However, other studies reported reduced [292, 311, 380] or no change in catalase activity after endurance exercise [293, 365].

*(5) GPx.* Athletes had higher activity than untrained subjects [352]. Also, physically active elders had higher activity than less active individuals in their erythrocytes [381]. Long term endurance exercise increased GPx activity in healthy adults [354, 355] and upregulated gene expression in congestive heart failure patients [361]. In experimental animals, increased activity was observed in heart [136, 356, 382, 383], liver [126, 136, 356, 372, 377, 382], lung [356, 377], kidney [136], brain [377, 378], testes [377], and skeletal muscles [311, 313, 371, 382–384], particularly in aged animals [126, 313, 356, 377, 378, 384]. Other investigators reported reduction [380] and no change [292, 293, 298] in GPx activity following endurance exercise. Increased GPx gene and protein expression following long term endurance exercise were also reported [307, 372].

*(6) GSR.* Increased activity was reported in humans [355] as well as in rats' brain, liver, lung, muscles, heart, muscle, and testes [311, 377, 382, 383]. Other studies reported that exercise training produced no change in GPx activity [292, 298].

*(7) GST.* Increased activity was reported in liver and salivary glands of mice after 8 weeks of treadmill training [136].

*(8) Thioredoxin Reductase 1.* Exercise increased thioredoxin reductase 1 (TrxR1), one of the thioredoxin system enzymes with direct and indirect antioxidant effects, in peripheral blood mononuclear cells in humans [205, 385].

*(9) NAD(P)H Oxidase.* Reduced gene [386], protein expression [194, 360, 365, 386, 387], and activity [359, 360] have been detected in humans [386] and experimental animals following endurance exercise. Also, the physically active elderly had lower NAD(P)H oxidase activity in their vascular endothelial cells compared with less active subjects [133].

Exercise-induced adaptation of antioxidant and prooxidant enzymes is highly isoform [296, 307, 370, 372, 373], tissue [311, 362, 371, 377, 378, 383], age [23, 138, 222, 297, 311, 366, 384], time course [23, 230, 292, 362], and exercise mode specific [352, 373, 388]. Exercise modulates the three SOD isoforms differently [296, 362, 365, 372, 373, 389] as the promoter region of SOD-2 contains more ROS-sensitive binding sites [390]. Exercise-induced protein expression of SOD is time dependent; SOD-1 protein expression was increased in rat skeletal muscles 48 hours after exercise, whereas SOD-2 protein content was increased after 10 and 24 hours but not 48 hours [362].

*Effects of Exercise on Repair Mechanisms.* Exercise can also stimulate the proteasome complex, which is responsible for the degradation of oxidatively damaged proteins [308, 391–393], and therefore enhances the cellular repair processes. Exercise modulates the activity of DNA repair enzymes, particularly oxoguanine DNA glycosylase (OGG1) and uracil DNA glycosylase (UDG), and thus reduces the accumulation of nuclear 8-hydroxydeoxyguanosine (8-OHdG) and mutations in skeletal muscles [314, 394, 395] but not brains of aged rats [396].

**Figure 4:**
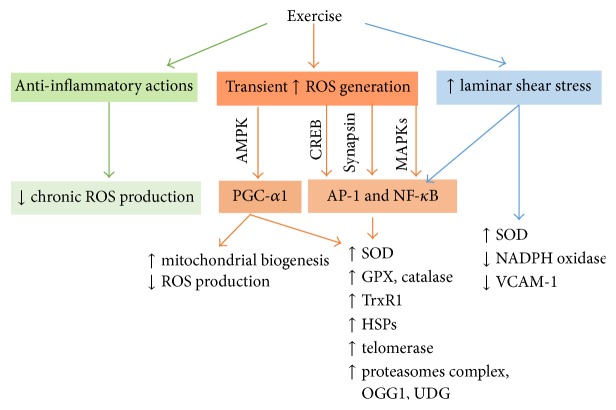
Signaling pathways underlying the antioxidant actions of exercise. AMPK = AMP-activated protein kinase, AP-1 = activator protein-1, CREB = cAMP-response-element binding, HSPs = heat shock proteins, GPX = glutathione peroxidase, MAPKs = mitogen activated protein kinases, NF-*κ*B = nuclear factor kappa B, OGG1 = oxoguanine DNA glycosylase, PGC-1*α* = peroxisome proliferator-activated receptor gamma, coactivator 1-alpha, SOD = superoxide dismutase, ROS = reactive oxygen species, TrxR1 = thioredoxin reductase 1, and UDG = uracil DNA glycosylase.

*Effects of Exercise on Telomeres.* Telomeres are often regarded as “the guardians of the genome.” Telomere dysfunction activates p53, leading to suppression of PGC-1*α* and PGC-1*β* promoters with consequent metabolic and organ failure [397]. Ten cross-sectional and longitudinal studies described a positive association of physical activity with telomere length in immune cells and skeletal muscles [398]. The leukocyte telomere was 200 nucleotides longer in people who exercise regularly, which roughly corresponds to a ten-year increase in longevity [399]. Exercise increases the activity of telomerase and induces the expression of telomere repeat-binding factor 2 and Ku70 in thoracic aorta and leukocytes from mice and humans [400]. However, other studies showed no association or inverted U relationship of physical activity with telomere length [398] warranting further investigation. The signaling pathways underlying the antioxidant actions of exercise are summarized in Figure 4.

Exercise training confers a myriad of physiological benefits in aging and cardiovascular diseases through its antioxidant and anti-inflammatory actions. The inflammatory actions of exercise are mainly exerted on adipose tissue (by reducing its mass and inflammatory environment), on the immune system (by shifting immune cells towards the less inflammatory phenotype, modulating the cytokines profile, and stimulating glucocorticoids), on skeletal muscles (by stimulating mitochondrial biogenesis, upregulating the anabolic myokine IL-15, anti-inflammatory cytokines, and repair proteins, improving muscle mass and strength, and reducing proinflammatory cytokines), and on the vasculature (by increasing laminar shear stress). It is likely that regular exercise exerts the most substantial anti-inflammatory effects in patients having high baseline inflammatory biomarkers, particularly when associated with visceral fat loss.

Exercise exerts antioxidant effects by suppressing inflammatory pathways and therefore inhibiting prominent sources of RONS generation. Importantly, exercise also activates redox-sensitive transcription factors, mainly NF-*κ*B and AP-1 and PGC-1*α*, leading to the enhancement of the antioxidant defense mechanisms by enhancing the expression and activities of SOD, catalase, GPx, GSR, GST, and TrxR1, while downregulating NADPH oxidase. Exercise also upregulates repair proteins such as HSPs, proteasome complex, OGG1, UDG, and telomerase. It is clear that the effects of exercise vary depending on the type, intensity, frequency, and duration of exercise, and also on the individual's age, sex, fitness level, health status, and endurance capacity. More integrative and innovative research approaches such as proteomics and metabolomics should be utilized to reveal the whole map of the molecular transducers of exercise benefits and risks not only at tissue/organ level but also at the whole organism level. This will allow the development of personalized exercise program and hold the promise for transformative discoveries of novel therapeutic targets.

## Figures and Tables

**Table 1:** Effect of physical activity/exercise on inflammatory mediators in the elderly.

Study	Mediator	Subjects	Tissue	Physical activity/exercise	Effect of physical activity/exercise	Reference
Observational	TNF-*α*	≥65 years *n* = 1004	Plasma	Self-reported physical activity	Inverse association between log TNF-*α* and physical activity	[249]
		65–80 years *n* = 30	Serum	Regular exercise	Lower percentage in the physically active subgroup	[250]
	CRP	≥65 years *n* = 5,888	Blood	Self-reported physical activity	Inverse association between physical activity and CRP	[241]
		Men, 58 years *n* = 391	Blood	Self-reported leisure time physical activity	Inverse association between physical activity and CRP	[242]
		70 to 79 years *n* = 870	Blood	Self-reported physical activity	Inverse association between CRP and physical activity	[244]
		70 to 79 years *n* = 3,075	Blood	Previous week exercise and physical activities	Inverse association between physical activity and CRP	[245]
		60 to 79 years *n* = 3810	Plasma	Self-reported physical activity	Inverse association between CRP and physical activity	[247]
		70 to 79 years *n* = 880	Plasma	Physical function measures included handgrip strength, signature time, chair stands, and 6-minute walk time	Inverse association between CRP and higher walking speed and grip strength	[248]
		≥65 years *n* = 1004	Plasma	Self-reported physical activity	Inverse association between and log CRP and physical activity	[249]
		65–80 years *n* = 30	Serum	Regular exercise	Lower level in the physically active subgroup	[250]
		50 to 70 years *n* = 3289	Plasma	Self-reported physical activity	Inverse association between CRP and physical activity	[195]
	IL-6	Men, 65–74 years *n* = 12	Serum	Self-reported physical activity	Lower levels of IL-6 in the physically active group	[246]
		70 to 79 years *n* = 3,075	Blood	Previous week exercise and physical activities	Lower level associated with higher level of physical activity	[245]
		70 to 79 years *n* = 870	Blood	Self-reported physical activities	Inverse association between IL-6 and physical activity	[244]
		≥65 years *n* = 1004	Plasma	Self-reported physical activity	Inverse association between log IL-6 and physical activity	[249]
		70 to 79 years *n* = 880	Plasma	Physical function measures included handgrip strength, signature time, chair stands, and 6-minute walk time	Inverse association between IL-6 and higher walking speed	[248]
	IL-10	Men, 65–74 years *n* = 12	Serum	Self-reported physical activity	Higher levels of IL-10 in the physically active group	[246]
	CD14+CD16+	65–80 years *n* = 30	Serum	Regular exercise	Lower percentage in the physically active subgroup	[250]
	Adiponectin	50 to 70 years *n* = 3289	Plasma	Self-reported physical activity	Direct association between adiponectin and physical activity	[195]

Interventional	TNF-*α*	≥64 years *n* = 105	Blood	Aerobic or flexibility/strength exercise for 10 months	Reduced level by aerobic and strength exercise	[266]
		Men, 67 ± 8 years with congestive heart failure *n* = 28	Plasma	Exercise training for 3 months	Level reduced after training	[267]
		81 ± 1 years *n* = 13	Skeletal muscle	Exercise training for 3 months	Reduced mRNA and protein levels after training	[268]
		65–80 years, physically inactive *n* = 15	Blood	3 days/week endurance and resistance exercise training for 12 weeks	Reduced level compared with pretraining values	[250]
		Postmenopausal women, 65–80 years *n* = 20	Blood	Regular exercise for previous 6 months	No change in protein or mRNA	[275]
		65–80 years *n* = 8	Serum	Progressive resistance strength training for 12 weeks	No change	[280]
	sTNF-R1	Overweight/obese sedentary with knee osteoarthritis ≥60 years *n* = 316	Serum	Combined weight training and walking for 1 h, 3 times/week for 18 months	No change	[277]
	CRP	Type 2 diabetic patients, >55 years *n* = 62	Serum	Strength training for 16 weeks	Reduced level after training	[196]
		>64 years *n* = 105	Blood	Aerobic or flexibility/strength exercise for 10 months	Reduced level by aerobic but not strength exercise	[266]
		Postmenopausal overweight or obese, sedentary women, 50–75 years *n* = 115	Serum	Moderate-intensity aerobic exercise for 12 months	Level reduced after training	[269]
		Women with the metabolic syndrome, 68.7 ± 3.4 years *N* = 32	Blood	Four sessions of high-intensity aerobic and resistance exercise per week for 12 months	Level reduced after training	[271]
		Patients with CHD, 66.7 ± 11 years *n* = 235 Controls 63.9 ± 11.1 years *n* = 42	Blood	Cardiac rehabilitation and exercise training for 3 months	Level reduced after training	[100]
		60 to 85 years *n* = 30	Serum	Exercise training for 6 months	No change	[276]
		Overweight/obese sedentary with knee osteoarthritis ≥60 years *N* = 316	Serum	Combined weight training and walking for 1 h, 3 times/week for 18 months	No change	[277]
		Postmenopausal breast cancer survivors, 50 to 69 years *n* = 52	Serum	Cycling 3 times/week for 15 weeks	No change	[278]
	IL-6	>64 years *n* = 105	Blood	Aerobic or flexibility/strength exercise for 10 months	Reduced level by aerobic but not strength exercise	[266]
		70–89 years *n* = 424	Plasma	Moderate-intensity combination of aerobic, strength, balance, and flexibility exercises for 12 months	Reduced IL-6 level but not CRP	[270]
		Young (20–30 years) and aged (66–76 years) *n* = 60	Blood	Endurance (20 min) and resistance exercise 3 days/week for 12 weeks	Stimulated level was reduced in young and old subjects	[263]
		Postmenopausal women, 65–80 years *n* = 20	Blood	Regular exercise for 6 months	No change in protein or mRNA	[275]
		Overweight/obese sedentary with knee osteoarthritis ≥60 years *N* = 316	Serum	Combined weight training and walking for 1 h, 3 times/week for 18 months	No change	[277]
		65–80 years *n* = 8	Serum	Progressive resistance strength training for 12 weeks	No change	[280]
	IL-1*β*	65–80 years *n* = 8	Serum	Progressive resistance strength training for 12 weeks	No change	[280]
	IL-18	>64 years *n* = 105	Blood	Aerobic or flexibility/strength exercise for 10 months	Reduced level by aerobic but not strength exercise	[266]
	TLR4	Postmenopausal women, 65–80 years	Blood	Regular exercise for 6 months	Lower level in trained versus untrained	[275]
		Young (20–30 years) and aged (66–76 years) *n* = 60	CD14+ cell	Endurance (20 min) and resistance exercise 3 days/week for 12 weeks	Level reduced in young and old subjects	[263]
	CD14+CD16+	65–80 years sedentary *n* = 15	Blood	Endurance and resistance exercise training for 12 weeks (3 days/week)	Reduced level compared with pretraining values	[250]
	Adiponectin	Type 2 diabetic patients >55 years *n* = 62	Serum	Strength training for 16 weeks	Increased level after training	[196]

**Table 2:** Effects of exercise training on HSPs in humans and experimental animals.

HSP	Species		Tissue	Physical activity/exercise mode	Effect of physical activity/exercise	References
HSP72	Human	Men, 22.1 ± 3.8 years *n* = 7	Plasma	Semirecumbent cycling for 120 min	Levels increased after exercise	[289]
		Men and women, 22–30 years *n* = 6	Serum	Acute bout of treadmill running for 60 min	Protein expression increased during and after exercise	[219]
		Men and women, 22–30 years *n* = 6	Skeletal muscle	Acute bout of treadmill running for 60 min	mRNA level increased after exercise	[219]
	Rats	Young (3 months) and aged (30 months)	Skeletal muscle	4.5 weeks of resistance exercise	Protein expression increased in young and old rats	[222]
		Adult females	Heart	1 or 3 consecutive days for 100 min at a speed of 20 m/min	Increased expression	[290]
		Adult males	Heart	Treadmill running for 1 or 3 days	Increased levels after 3 but not 1 day	[291]
		Adult males	Heart	24-week but not 12-week treadmill training	Increased expression	[292]
		Females, 4 months	Heart	Endurance exercise for 10 weeks	Increased expression	[293]
		Females, 4 months	Ventricle	3–5 consecutive days of treadmill exercise [60 min/day at 60–70% maximal O_2_ uptake]	Increased levels	[368]

HSP70	Human	Male athletes, 32.3 ± 9.3 years *N* = 12	Leukocytes	Half marathon run	Protein expression increased	[294]
		Men and women, 26 ± 4 years *N* = 5	Skeletal muscle	30 min on a treadmill	mRNA level but not protein level increased at 4 min, 30 min, and 3 h after exercise	[221]
		Women, 22 ± 2.2 years *n* = 10	Skeletal muscles	Acute bout of eccentric contractions	Protein expression increased after exercise	[218]
	Rats	Aged 24 months old	Hearts	Treadmill training for 30 m/min, 45 min/day, 5 days/week for 6 weeks	Expression increased	[295]
		Young (6 months) and aged 27 months	Left ventricle	Treadmill for 60 min/day, 5 days/week for a total of 12 weeks	Protein increased in the young group compared with sedentary control	[296]
		Males, young (4 months) and aged (21 months)	Heart	Acute exercise for 60 min at 70–75% of maximum oxygen consumption	Expression increased in young and old rats	[297]
		Males, 2 months	Heart	Treadmill for 3 days/week for 14 weeks	Increased protein level	[137]
		Spontaneously hypertensive females (9 weeks)	Aorta	Voluntary wheel running for 5 weeks	Reduced gene expression	[299]
		Males and females, 11 weeks	Skeletal muscle	Acute treadmill running for 30 min	Protein and mRNA expression increased in males but not females	[220]
	Mice	Males (6–8 weeks)	Cardiac ventricles	Swimming training for 14 weeks	No change	[298]

HSP60	Human	Male athletes, 32.3 ± 9.3 years *N* = 12	Leukocytes	Half marathon run	Expression increased	[294]
	Rats	Males, 2 months	Heart	Treadmill for 3 days/week for 14 weeks	Decreased mRNA	[137]
		Spontaneously hypertensive females (9 weeks)	Aorta	Voluntary wheel running for 5 weeks	Reduced gene expression	[299]
	Mice	Males, 6–8 weeks	Ventricles	Swimming training for 14 weeks	Increased level	[298]

HSP32	Rats	Females, 4 months	Heart	Endurance exercise for 10 weeks	No change	[293]

HSP27	Human	Male athletes, 32.3 ± 9.3 years *N* = 12	Leukocytes	Half marathon run	Expression increased	[294]
	Rats	Males, aged (24 months)	Hearts	Treadmill training for 30 m/min, 45 min/day, 5 days/week for 6 weeks	Expression increased	[295]

HSP25	Rats	Males, young (3 months) and aged (30 months)	Skeletal muscles	4.5 weeks of resistance exercise	Protein expression increased in young and old rats	[222]

HSC70	Human	Male athletes, 32.3 ± 9.3 years *N* = 12	Leukocytes	Half marathon run	No change	[294]
	Rats	Males, young (3 months) and aged (30 months)	Skeletal muscles	Resistance exercise for 4.5 weeks	No change in protein expression	[222]

**Table 3:** Effects of exercise training on expression and activity of antioxidant and prooxidant enzymes.

Enzyme	Species		Tissue	Exercise mode	Effect of physical activity/exercise	Reference
SOD	Human	Untrained males *n* = 9	Erythrocytes	High-intensity endurance training for 12 weeks	Activity increased after training	[354]
		Healthy young men and women *n* = 17	Erythrocytes	16 weeks of training then an acute bout of aerobic exercise for 30 min	Transient increase in activity after acute exercise	[355]
		Athletes *n* = 18 and sedentary control *n* = 6	Erythrocyte	Marathon or sprint training	Higher activity in sprint-trained athletes and marathon runners	[352]
		Winter swimmers *n* = 40 and controls *n* = 36	Erythrocyte	Regular winter swimming	Higher activity in winter swimmers	[353]
	Rats	Males, young and aged (17 months)	Lung, heart, and liver	Regular swimming exercise for 1 year	Increased activity in lung and heart of old rats relative to sedentary controls	[356]
		Males, young and aged	Heart	Treadmill endurance exercise for 2 months	Increased activity in young and aged rats	[316]
		Male, young, adult, and aged	Skeletal muscles	Exercise training for 10 weeks	Increased activity in deep vastus lateralis muscle of young rats only	[311]
		Males, 16-17 weeks	Heart and skeletal muscle	Sprint training on a treadmill for 6 weeks	Unchanged activity	[383]
		Females, 4 months	Ventricles	Endurance exercise training for 10 weeks	Increased activity	[293]
		Females, 17 weeks	Ventricles	High-intensity exercise treadmill for 10 weeks	Increased activity	[357]
		Males, adults	Heart	Treadmill training for 12 or 24 weeks	Increased activity after 24 but not 12 weeks	[292]
		Males, myocardial infarcted	Aorta	Treadmill training 5 times per week, 60 min/day for 11 weeks	Increased activity but not expression	[359]
	Mice	Males, aged 29–32 months	Aorta	Voluntary wheel running for 10–14 weeks	Increased activity	[360]
		Males and females, aged 28, 52, and 78 weeks	Bain, heart, liver, and kidney	Long term moderate-intensity treadmill exercise	Increased activity in all tissues at 52 but not 78 weeks old	[23]
		Females, 3 months	Kidney, heart, and skeletal muscle	Treadmill exercise for 8 weeks	Increased activity	[136]
		Males (6–8 weeks)	Ventricles	Swimming training for 14 weeks	No change in activity	[298]
	Microtus	Short-tailed field vole *Microtus agrestis*	Skeletal muscle and heart	Voluntary running over 1 or 7 days	Reduced activity in the heart	[401]

SOD-1	Human		Umbilical vein endothelial cells	Laminar fluid shear stress	mRNA and protein levels increased after 24 hours	[350]
			Endothelial progenitor cells	Shear stress	Increased mRNA expression and activity	[349]
			Aortic endothelial cells	Fluid shear stress	Increased protein expression	[351]
		Patients with congestive heart failure *n* = 14	Skeletal muscle	12 weeks of training	Increased gene expression	[361]
	Rats	Young (3 months) and aged (30 months)	Skeletal muscles	4.5 weeks of resistance exercise	Protein expression increased in young rats but decreased in old rats	[222]
		Males, young (3–5 months) and aged (24–27 months)	Skeletal muscles and heart	Exhausting treadmill running	Increased activity in skeletal muscles and heart of young rats and hearts of old rats	[358]
		Males, aged 24 months	Hearts	Treadmill training 30 m/min, 45 min/day, 5 days/week for 6 weeks	Protein expression increased	[295]
		Males, young (2 months) and old (22 months)	Soleus muscle feed arteries	Exercise training for 10–12 weeks	No change in protein expression	[366]
		Females, 12 months old	Hippocampus	Treadmill training for 15 weeks	Protein expression increased	[307]
		Females	Skeletal muscle	Acute bout of exhaustive treadmill exercise	Increased protein level but not mRNA or activity	[362]
		Males, adults	Heart	Acute session of treadmill running for 25–30 min	No change	[230]
		Females, adults	Ventricles	20 weeks of training	Increased protein expression	[363]
		Males, high caloric fed	Aorta and mesenteric artery	Running 60 min, 5 days/week for 12 weeks	Increased expression relative to sedentary controls	[364]
	Mice		Aorta	Treadmill running 15 m/min, 30 min/day, 5 days/week for 3 weeks	No change in protein expression	[367]
		Males, diabetic young	Aorta	Treadmill exercise for 10 weeks	Increased protein expression	[194]
	Pigs	Females	Aortic endothelial cells	Chronic exercise training for 16–19 weeks	Protein and activity increased	[365]

SOD-2	Human	Men *n* = 18	Plasma	Swimming or running for 3 months then a bout of acute exercise	Protein level increased by acute exercise	[373]
		Men 62 ± 3 years Physically active *n* = 13 and sedentary *n* = 26	Vascular endothelial cells from the brachial artery	Habitual aerobic exercise	Higher protein expression than sedentary men	[133]
	Rats	Males, 10-11 weeks	Cardiac mitochondria	Long term voluntary wheel running	Reduced activity	[402]
		Males, adults	Heart	Treadmill training for 12 or 24 weeks	Increased activity after 24 but not 12 weeks	[292]
		Males, adults	Heart	Acute session of treadmill running for 25–30 min	Activity increased at 0.5 and 48 h, and protein content increased at 48 h after exercise	[230]
		Females, 4 months	Heart	3–5 consecutive days of treadmill exercise [60 min/day at 60–70% maximal O_2_ uptake]	Increased activity	[368]
		Females, 4 months	Ventricles	Treadmill exercise (60 min/day) at 25 degrees for 3 days	Increased activity	[369]
		Females, adults	Ventricles	20 weeks of training	Increased protein expression	[363]
		Males subjected to IR	Ventricles	3 consecutive days of intensive treadmill exercise 60 min/day, at 30 m/min	Increased activity of SOD-2 but not SOD-1	[370]
		Males, aged 24 months	Heart	Treadmill training 30 m/min, 45 min/day, 5 days/week for 6 weeks	Protein expression increased	[295]
		Males, young (4 months) and aged (21 months)	Heart	Acute exercise 60 min at 70–75% of maximum oxygen consumption	Activity increased in old rats	[297]
		Young (6 months) and aged 27 months	Left ventricle	Treadmill for 60 min/day, 5 days/week for a total of 12 weeks	Protein expression and activity increased in the aged group compared with sedentary control	[296]
		Females	Skeletal muscle	Treadmill running for 10 weeks	Increased activity and protein expression	[371]
		Females	Skeletal muscles	Acute bout of exhaustive treadmill exercise	Increased mRNA level in deep vastus lateralis muscle. Increased protein level in superficial vastus lateralis	[362]
		Male Zucker diabetic fatty rats (18 weeks)	Skeletal muscles	Swimming training for 6 weeks	Protein expression increased	[375]
		Males, 2 months	Plasma	Treadmill training 3 days/week for 14 weeks	Increased activity	[137]
		Males, obese Zucker	Liver	Treadmill running at 20 m/min for 1 h/day, 7 days/week, for 8 weeks	mRNA and protein levels and activity increased	[372]
		Male, young (3 months) and aged (23 months)	Aorta	Treadmill training for 12 weeks	Increased protein expression in aged rats	[138]
	Mice	Male, diabetic and young	Heart	Motorized exercise-wheel for 1 h/day, 5 days/week for 8 weeks	Increased protein expression	[374]
		Male, diabetic and young	Aorta	Motorized exercise-wheel for 1 h/day, 5 days/week for 8 weeks	Increased protein expression	[376]
	Pigs	Females	Aortic endothelial cells	Chronic exercise training for 16–19 weeks	No change in protein levels	[365]

SOD-3	Human	Men 62 ± 3 years Physically active *n* = 13 and sedentary *n* = 26	Vascular endothelial cells from the brachial artery	Habitual aerobic exercise	Higher activity than sedentary men	[133]
		Men *n* = 18	Plasma	Swimming or running for 3 months then a bout of acute exercise	Reduced protein level after endurance training but increased by acute exercise	[373]
	Rats	Males, young (2 months) and old (22 months)	Soleus muscle feed arteries	Exercise training for 10–12 weeks	Increased protein expression in old rats	[366]
	Mice		Aorta	Treadmill running 15 m/min, 30 min/day, 5 days/ week for 3 weeks	Increased protein expression	[367]

CAT	Human	Athletes *n* = 18 and sedentary control *n* = 6	Erythrocytes	Marathon or sprint training	Lower activity than controls in sprint-trained athletes	[352]
		Winter swimmers *n* = 40 and controls *n* = 36	Erythrocytes	Regular winter swimming	Higher activity in winter swimmers	[353]
	Rats	Males, young and aged (17 months)	Lung, heart and liver	Regular swimming exercise for 1 year	Increased activity in liver of old rats relative to sedentary controls	[356]
		Aged	Brain, liver, lung, muscle, and testes	Regular exercise	Increased activity in all tissues	[377]
		Males, young (4 months) and aged (21 months)	Heart	Acute exercise 60 min at 70–75% of maximum oxygen consumption	Activity increased in young and old rats	[297]
		Male, young and aged	Heart	Treadmill exercise for 2 months	Increased activity in young and aged rats	[316]
		Males, young (9 months) and aged (20 months)	Liver	Regular exercise	Increased activity	[126]
		Male, young (8 months) and aged (22 months)	Brain	Swimming 30 min/day, 5 days/week for 12 weeks	Increased activity in hippocampus in young and old rats	[378]
		Male, young (8 weeks), adult (12 months), and old (24 months)	Skeletal muscles	Exercise training for 10 weeks	Decreased activity in soleus muscle of adult and old rats	[311]
		Males (4 months)	Cardiac mitochondria	Treadmill for 16 weeks (5 days/week, 60 min/day, 25 m/min)	Increased activity	[379]
		Males, adults	Heart	Treadmill training for 12 or 24 weeks	Reduced activity after 24 but not 12 weeks	[292]
		Females, 4 months	Ventricles	Endurance exercise training for 10 weeks	No change in activity	[293]
		Males subjected to IR	Ventricles	3 consecutive days of intensive treadmill exercise 60 min/day, at 30 m/min	Increased activity	[370]
		Females	Skeletal muscle	Treadmill running for 10 weeks	Increased activity in deep vastus lateralis muscle	[371]
		Male, normotensive and hypertensive (11-12 weeks)	Liver, kidney, skeletal muscles, and heart	Treadmill running for 10 weeks	Reduced activity in all tissues in hypertensive and normotensive rats	[380]
	Mice	Males and females, aged 28, 52, and 78 weeks	Brain, heart, liver, and kidney	Long term moderate-intensity treadmill exercise	Increased activity in all issues at 52- but not 78-week-old mice	[23]
		Females, 3 months	Liver, heart, skeletal muscle, and salivary gland	Treadmill for a total of 8 weeks	Increased activity	[136]
	Pigs	Females	Aortic endothelial cells	Exercise training for 16–19 weeks	No change in protein level	[365]

GPX	Human	Untrained males *n* = 9	Erythrocytes	High-intensity endurance training for 12 weeks	Increased activity after training	[354]
		Healthy young *n* = 17	Blood	16 weeks of training then an acute bout of aerobic exercise for 30 min	Activity increased after regular training and transiently reduced following acute exercise	[355]
		Exercising and sedentary, young (21–38 years) and old (65–75 years) *n* = 50	Erythrocyte	Regular exercise	Higher activity in the exercising elderly compared to the sedentary elderly	[381]
		Athletes *n* = 18 and sedentary control *n* = 6	Erythrocyte	Marathon or sprint training	Higher activity in sprint-trained athletes	[352]
		Patients with CHF *n* = 14	Skeletal muscle	Moderate-intensity semirecumbent bicycle training for 12 weeks	Increased gene expression	[361]
	Rats	Males, young and aged (17 months)	Lung, heart, and liver	Regular swimming for 1 year	Activity increased in liver, lung, and heart of old rats relative to sedentary controls	[356]
		Aged	Brain liver, lung, muscle, and testes	Regular exercise	Increased activity in brain, liver, lung, and testes	[377]
		Male, young (8 months) and aged (22 months)	Brain	Swimming 30 min/day, 5 days/week for 12 weeks	Increased activity in hippocampus in young and aged	[378]
		Females, 12 months old	Hippocampus	Treadmill for a period of 15 weeks	Protein expression increased	[307]
		Females (24 months)	Skeletal muscles	Treadmill training (60 min, 5 days/week for 10 weeks)	Increased activity	[313]
		Male, young (8 weeks), adult (12 months), and old (24 months)	Skeletal muscles	Exercise training for 10 weeks	Increased activity in deep vastus lateralis muscle of young rats only	[311]
		Young (5 months) and aged (27.5 months)	Skeletal muscles	Treadmill training for 10 weeks	Increased activity in deep vastus lateralis muscle of old rats only	[384]
		Males, young (9 months) and aged (20 months)	liver	Regular exercise	Increased activity	[126]
		Males, obese Zucker	Liver	Treadmill running at 20 m/min for 1 h/day, 7 days/week for 8 weeks	mRNA and protein levels and activity increased	[372]
		Males, young	Liver, heart, and muscle	Swim training for 10 weeks	Increased activity in all tissues	[382]
		Females, 4 months	Ventricles	Endurance training for 10 weeks	No change in activity	[293]
		Males, adult	Heart	Treadmill training for 12 or 24 weeks	No change in activity	[292]
		Males, 16-17 weeks	Skeletal muscle and heart	Sprint training on a treadmill for 6 weeks	Increased activity in heart and some skeletal muscle fibres	[383]
		Females	Skeletal muscle	Treadmill running for 10 weeks	Increased activity in deep vastus lateralis muscle	[371]
		Male, normotensive and hypertensive (11-12 weeks)	Liver, kidney, skeletal muscles, and heart	Treadmill running for 10 weeks	Reduced activity in all tissues in hypertensive and normotensive rats	[380]
	Mice	Females, 3 months	Liver, kidney, and heart	Treadmill for a total of 8 weeks	Increased activity	[136]
		Males (6–8 weeks)	Ventricles	Swimming training for 14 weeks	No change in activity	[298]

GSR	Human	Healthy young *n* = 17	Plasma	16 weeks of training then an acute bout of aerobic exercise for 30 min	Activity increased after regular training	[355]
	Mice	Males (6–8 weeks)	Ventricles	Swimming training for 14 weeks	No change in activity	[298]
	Rats	Males, adults	Heart	Treadmill training for 12 or 24 weeks	No change in activity	[292]
		Males, 16-17 weeks	Skeletal muscle and heart	Sprint training on a treadmill for 6 weeks	Increased activity in heart and some skeletal muscle fibres	[383]
		Males, young	Liver, heart, and muscle	Swim training for 10 weeks	Increased activity in all tissues	[382]
		Aged	Brain liver, lung, muscle, and testes	Regular exercise	Increased activity in testes	[377]
		Males, young (8 weeks), adult (12 months), and old (24 months)	Skeletal muscles	Exercise training for 10 weeks	Activity increased in deep vastus lateralis muscle of young rats and decreased in soleus muscle of adult rats only	[311]

GST	Mice	Females, 3 months	Liver and salivary gland	Treadmill for a total of 8 weeks	Increased activity	[136]

NAD(P)H oxidase	Human	Patients with symptomatic coronary artery disease *n* = 45	Internal mammary artery	Aerobic training for 4 weeks	Reduced protein and gene expression and activity	[386]
		Men, 62 ± 3 years, physically active *n* = 13 and sedentary *n* = 26	Vascular endothelial cells from the brachial artery	Habitual aerobic exercise	Lower level of p47(phox) compared with sedentary men	[133]
	Rats	Males, young and myocardial infarcted	Aorta	Treadmill training 5 times per week, 60 min/day for 11 weeks	Reduced activity	[359]
		Male, adult (6 months) and aged (24 months)	Aorta	Swim training (60 min/day, 5 days/week for 10 weeks)	Decreased expression of gp91(phox)	[387]
	Pigs	Females	Aortic endothelial cells	Chronic exercise training for 16–19 weeks	Reduced protein expression of p67(phox)	[365]
	Mice	Young (6–8 months) and aged (29–32 months)	Aorta	Voluntary wheel running for 10–14 weeks	Reduced expression and activity	[360]
		Males, diabetic and young	Aorta	Treadmill exercise for 10 weeks	Decreased protein expression of gp91(phox)	[194]
